# Protocol for the pilot quasi-experimental controlled trial of a gender-responsive implementation strategy with providers to improve HIV outcomes in Uganda

**DOI:** 10.1186/s40814-022-01202-0

**Published:** 2022-12-23

**Authors:** K. M. Sileo, R. K. Wanyenze, A. Anecho, R. Luttinen, C. Semei, B. Mukasa, W. Musoke, S. H. Vermund, S. L. Dworkin, J. F. Dovidio, B. S. Taylor, T. S. Kershaw

**Affiliations:** 1 Department of Public Health, The University of Texas at San Antonio, San Antonio, TX USA; 2 Department of Disease Control and Environmental Health, Makerere University School of Public Health, Kampala, Uganda; 3 Department of Demography, The University of Texas at San Antonio, San Antonio, TX USA; 4 Mildmay Uganda, Kampala, Uganda; 5 Yale School of Public Health, New Haven, CT USA; 6 School of Nursing and Health Studies, University of Washington Bothell, Bothell, WA USA; 7 Department of Psychology, Yale University, New Haven, CT USA; 8 Division of Infectious Diseases, Department of Medicine, Joe R. & Teresa Lozano Long School of Medicine, UT Health San Antonio, San Antonio, TX USA; 9 Department of Social and Behavioral Sciences, Yale School of Public Health, New Haven, CT USA

**Keywords:** HIV/AIDS, Intervention, Implementation science, Uganda

## Abstract

**Background:**

Antiretroviral treatment (ART) is the most effective clinical intervention for reducing morbidity and mortality among persons living with HIV. However, in Uganda, there are disparities between men and women in viral load suppression and related HIV care engagement outcomes, which suggests problems with the implementation of ART. Gender norms are a known driver of HIV disparities in sub-Saharan Africa, and patient-provider relationships are a key factor in HIV care engagement; therefore, the role of gender norms is important to consider in interventions to achieve the equitable provision of treatment and the quality of ART counseling.

**Methods:**

The overall research objective of this study is to pilot test an implementation strategy (i.e., methods to improve the implementation of an evidence-based intervention) to increase providers’ capacity to provide gender-responsive treatment and counseling to men and women on HIV treatment in Uganda. Delivered to HIV providers, this group training adapts evidence-based strategies to reduce gender biases and increase skills to deliver gender-specific and transformative HIV counseling to patients. The implementation strategy will be piloted through a quasi-experimental controlled trial. Clinics will be randomly assigned to either the intervention or control conditions. The trial will assess feasibility and acceptability and explore barriers and facilitators to implementation and future adoption while gathering preliminary evidence on the implementation strategy’s effectiveness by comparing changes in patient (*N* = 240) and provider (*N* = 80–140) outcomes across intervention and control clinics through 12-month follow-up. Quantitative data will be descriptively analyzed, qualitative data will be analyzed through thematic analysis, and these data will be mixed during the presentation and interpretation of results where appropriate.

**Discussion:**

This pilot intervention trial will gather preliminary evidence on the acceptability, feasibility, and potential effect of a novel implementation strategy to improve men and women’s HIV care engagement, with the potential to reduce gender disparities in HIV outcomes.

**Trial registration:**

Clinicaltrials.gov NCT05178979, retrospectively registered on January 5, 2022

**Supplementary Information:**

The online version contains supplementary material available at 10.1186/s40814-022-01202-0.

## Background

Antiretroviral therapy (ART) is the single most effective clinical intervention in reducing the morbidity and mortality from HIV infection and reducing the risk of transmission of the virus to others. ART, when properly administered and consumed, extends the life years of infected individuals by decades and may reduce population HIV incidence by reducing infectiousness of treated persons [1, 2]. Coverage of ART in Uganda has tripled since 2010, resulting in a 45% reduction in HIV-related deaths [3]. Eighty-two percent of people living with HIV were virally suppressed in 2020 [3], but men on ART are less likely to be virally suppressed compared to women [4–6]. These disparities are in part explained by differences in patient behavior, with lower ART adherence and retention in care among men [7–10], for which gender norms play a central role [11, 12]. Norms asserting that men should be self-reliant, strong, and emotionally inexpressive amplify HIV stigma [13–21], leading to poor HIV care engagement [11, 12, 22, 23]. As such, the onus of responsibility for change has mostly been at the individual level; however, there is also evidence that gender inequities embedded in the broader health system influence HIV outcomes [24–26]. More research is needed that critically examines the delivery of health services to inform institutional interventions to reduce gender disparities in the provision of HIV treatment.

**Fig. 1:**
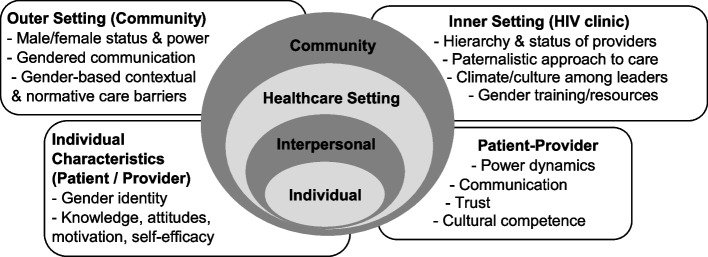
Social ecological model of gender and patient-provider relationships

Implementation science can provide methods and frameworks to progress towards this goal [27]. Implementation frameworks such as the Consolidated Framework for Implementation Science Research (CFIR) [28] can broaden our understanding of multi-level factors driving gender disparities by focusing on factors beyond the individual that affect the equitable delivery and quality of ART counseling [27]. Moreover, implementation strategies can be developed to reduce gender disparities (i.e., methods to enhance the implementation of a clinical intervention) [27, 29]. CFIR outlines 5 domains (intervention characteristics, outer setting, inner setting, characteristics of individuals, process) and 39 sub-domains that can affect implementation [28]. Based on the findings from a preliminary needs assessment conducted in Ugandan health facilities [30] and literature from Uganda and similar settings (discussed next), Fig. 1 maps CFIR constructs onto the social ecological model, a framework that emphasizes the interaction of factors across levels [31], to demonstrate how gender norms in the outer (community) and inner setting (health system) shape provider biases (characteristics of individuals) and patient-provider relationships (interpersonal) in ways that influence patient adherence and retention [32, 33]. This protocol proposes the evaluation of a gender-responsive implementation strategy (a training intervention for HIV providers) aimed to reduce gender disparities in ART outcomes by focusing on the patient-provider relationship, guided by CFIR and in synergy with the social ecological model.

### Gender-responsive HIV programs

There is wide recognition of the importance of gender-responsive HIV services at the health-system level (inner setting) to address HIV gender disparities for both women and men [24, 34]. The degree to which health programs address gender norms/inequity has been conceptualized on a spectrum [35, 36]. On one end, programs can perpetuate gender inequities (gender unequal) or ignore gender norms (gender blind/neutral). In the middle of the spectrum are those that acknowledge gender differences but do not address gender inequities (gender sensitive). On the other end of the spectrum are health programs that accommodate gender norms and consider women’s and men’s specific needs (gender specific) and those that address the cause of gender-based health inequities and work to transform harmful gender roles, norms, and relations (gender transformative).

Gender-responsive HIV services often require gender-sensitive training with health workers to improve their understanding and treatment of patients through education and content to help them reflect on and modify their own assumptions and biases based on gender and other intersecting identities [37]. These trainings can have positive effects on providers’ knowledge, attitudes, and skills [38]. Gender-specific programming tailored to women and men’s needs is also critical for reducing HIV gender disparities, as women’s and men’s experiences of HIV infection are unique [39]. For example, women-specific HIV services are multidimensional, often including the integration of services to meet multiple medical and social needs, inclusion of peer and social support services, and tailored counseling and services to address their unique barriers to HIV prevention and care, such as gender-based violence [37]. Although HIV programs similarly tailored to address men’s specific needs is needed and recognized [24], the health system’s historically gendered organization has systematically excluded men, resulting in a lack of HIV services tailored to their needs [25, 26].

Ideally, HIV programs go beyond being gender sensitive and specific to also be gender transformative. The World Health Organization defines gender-transformative approaches as those “that address the causes of gender-based health inequities through approaches that challenge and redress harmful and unequal gender norms, roles, and power relations that privilege men over women.” [40] There is strong evidence that gender-transformative programs are effective at improving HIV outcomes [41–44]. Recent systematic reviews that examine a gender-transformative approach within the context of sexual and reproductive health provide evidence supporting programs that employ multicomponent activities that target behavior change, approaches that work with *both* women and men, delivery of activities by trained facilitators over a sufficient period of time, and multilevel programming that mobilizes the wider community [43, 45]. However, most programs tend to focus on change at the individual and couple level, working directly with individual patients to alter their gender attitudes and relationships; institutional-level components, such as provider training or policy change, are less frequently included but could have a more sustained and wider impact [41, 43, 45, 46].

### Focusing on HIV providers and gendered aspects of the patient-provider relationship

Healthcare workers play a critical role in delivering gender-transformative programming [40, 47–50]. However, despite complexities in implementation [51], providers are seldom the focus of gender-transformative intervention research. Gender-sensitive and specific training with providers is a necessary precursor to build capacity among providers to deliver gender-transformative programming. In order to respond to gender disparities affecting HIV care engagement among patients, providers need to be aware of disparities, skilled and motivated to respond to them, and conscience of how their own biases and gender inequitable attitudes might affect their provision of HIV care. Providers’ empathy and treatment towards patients are shaped by HIV stigma and broader biases and stereotypes about HIV, which are gendered. For example, across sub-Saharan African contexts, there are differential, context-specific judgements for risk behavior, alcohol use, and infidelity for men and women, shaped by broader gender norms [32, 52, 53]. The stigmatization that patients encounter in clinical care is a known barrier to adherence [54], especially for newly diagnosed/ART-initiated patients [54–56]. These biases can shape providers’ perception and response to patients’ gendered barriers to HIV care, affecting ART counseling. For example, in one study, providers were more empathetic to men’s adherence barriers (e.g., work responsibilities) than women’s (e.g., childcare), resulting in the misconception that men had better adherence than women [32].

Other elements of the patient-provider relationship that are known determinants of patients’ ART adherence and retention in HIV care are those central to patient-centered care (e.g., communication, equitable decision-making, trust, cultural competence of providers); these relationship factors are also shaped by broader gender norms and may need to be intervened on [37, 57–61]. Inner setting/health system factors, such as status afforded to providers based on their occupation, a hierarchical culture of medicine, a paternalistic approach to care, and the culture and resources supporting gender equity within the health system, intersect with gender dynamics to undermine equitable power dynamics and reduce patient satisfaction with care [30, 32, 62]. For example, men describe being in the submissive role of patient as demasculinizing, particularly with female providers [11], which can be worsened by poor provider communication [63–67] and norms that men should be emotionally inexpressive [18, 68–70]. For women, norms from the outer/community setting that dictate women’s inferior sociocultural and economic status to men, indirect communication style, and deem discussions about sex taboo affect women’s care [30, 32]; for example, by constraining women’s ability to speak openly about topics related to HIV prevention and care, especially to male providers [32]. Indirect communication can influence providers’ gender biases, such as believing women are deceitful about their adherence and compromise patient-provider trust [32]. Distrust of providers is also a major barrier to men’s engagement, tied to concerns about confidentiality and HIV stigma [71].

### Reducing HIV gender disparities through gender-responsive training with HIV providers

Gender-responsive training that increases HIV providers’ awareness of how gender norms affect HIV care engagement for men and women, reduces their gender biases, and gives them skills to implement gender-sensitive, patient-centered communication skills, and gender-specific and gender-transformative approaches to counseling men and women could improve elements of the patient-provider relationship highlighted above (equity, communication, bias, trust, gender competence) that affect patient’s HIV care engagement. A large body of literature on provider-focused “cultural competency” interventions exists from resource-rich settings that can inform provider-level strategies to address elements of the patient-provider relationship shaped by gender norms in the Ugandan context. Drawing on evidence from a variety of settings with medical professionals, Dovidio and colleagues [72] have shown that increasing provider motivation [73–76], awareness [77–79], skills [80–83], empathy [84–89], and emotional regulation [90–93] about health disparities can prevent implicit racial biases from affecting clinical judgment and behavior, which they developed into a set of strategies intended to inform provider trainings [72]. These strategies could be adapted to be gender responsive (including gender-sensitive, specific, and transformative content), focused on addressing gender disparities in HIV care.

Cultural competency interventions aimed to reduce provider bias and improve cultural competence support the potential for provider trainings to improve patient-level outcomes, with evidence of moderate effects on provider outcomes (e.g., knowledge, attitudes), patient healthcare utilization (e.g., retention, adherence), some effect on clinical outcomes, and improvements in intermediate factors in the patient-provider relationship highlighted above (e.g., communication skills) [33, 94, 95]. However, the cultural competency literature also highlights a need for more rigorous evaluations to better assess clinical effects, trainings informed by theory, and more robust trainings (i.e., beyond single-/low-intensity sessions) with at least 12-month follow-up evaluation [94–97].

### Specific aims

**Fig. 2:**
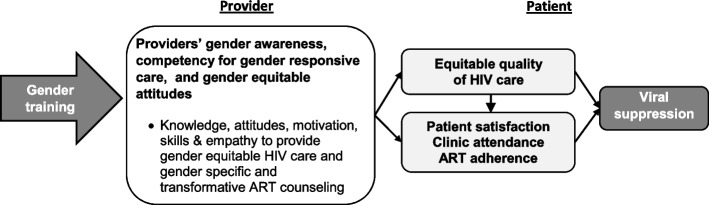
Conceptual model of the implementation strategy’s intended effect on provider and patient outcomes

Gender disparities that exist in HIV outcomes suggest a problem with the implementation of ART counseling. Gender norms shape patient-provider relationships in several ways that may contribute to this problem, but if appropriately informed and guided, providers could contribute to a solution that alleviates these disparities. This project aims to test a gender-responsive provider training that adapts an existing set of strategies [72] grounded in social cognitive psychology principles to be gender sensitive and specific by increasing provider knowledge, motivation, skills, and empathy to provide equitable care to women and men and gender transformative by altering providers’ own gender inequitable attitudes and providing them with skills to provide gender-transformative counseling to patients. The ultimate goal of the training is to improve the quality of HIV care delivered, increasing patient satisfaction, retention, and ART adherence and reducing gender disparities in HIV outcomes (see Fig. 2 for conceptual model).

This protocol describes the plans for a quasi-experimental, pre/post intervention design that will be used to pilot test a multi-session group training implementation strategy with HIV providers. The primary aims of the trial are to gather data on implementation outcomes related to feasibility and acceptability of the intervention (aim 1) and of the trial procedures (aim 2); CFIR will be used to inform the measurement of feasibility, acceptability, and other implementation outcomes for the intervention trial. An exploratory aim of this trial is to pilot test the implementation strategy’s preliminary effectiveness at improving newly diagnosed/unsuppressed patient outcomes related to HIV care engagement and HIV provider outcomes related to their gender awareness and competence to deliver gender-responsive care compared to control (no intervention) (aim 3). The overall objective and specific research aims are detailed below.

#### **Overall objective**

A quasi-experimental, pre/post intervention trial with a mixed-method process evaluation will assess feasibility, acceptability, and barriers/facilitators to implementation and future adoption (aim 1), the feasibility of trial procedures (aim 2), and compare the potential effectiveness of the implementation strategy at improving provider and patient outcomes (exploratory aim 3) compared to usual care.

- *Specific aim 1*: Using qualitative methods, explore the implementation strategy’s feasibility, acceptability, and barriers and facilitators to the training sessions’ implementation and potential for future adoption.
- *Specific aim 2*: Using process data, assess the feasibility of trial procedures (i.e., clinic mobilization, recruitment, retention, outcome measurement).
- *Exploratory aim 3*: Explore the implementation strategy’s preliminary effects by comparing changes in provider (N~80–140) and patient (*N* = 240) outcomes between intervention and control across baseline, 6, and 12 months.

## Methods

### Setting

This study will be carried out in partnership with Mildmay Uganda, a community-based organization (CBO). The pilot will be implemented at four governmental public health facilities  in two districts in central Uganda. In each district, to reach the target sample size, we anticipate needing a Health Centre V (general hospital) and Health Centre IV facility, based on Uganda's decentralized healthcare system ranging from Health Centre I to V. The study sites randomized to different conditions for the pilot trial will be at least 1 h apart (patients are unlikely to travel between sites and providers will work only at their respective clinic) to minimize the risk of contamination. Clinics will be selected based on similarities in facility-level, demographics (e.g., semi-rural), number of HIV patients, number of HIV providers (~20–35 per clinic and tend to have more women providers than men), immediate ART initiation, and routine viral load monitoring.

### Study design

#### Mixed methods: four-site quasi-experimental controlled intervention trial

This study employs a mixed-methods embedded experimental design, following Creswell and Plano-Clark [98]. This design included qualitative formative work to inform the refinement of the intervention (not reported in this protocol) [30], followed by a multi-site quasi-experimental controlled trial to test the intervention’s implementation and potential effects on patient and provider outcomes, and concluding with additional post intervention qualitative assessments used to understand the intervention’s feasibility and acceptability.

**Fig. 3:**
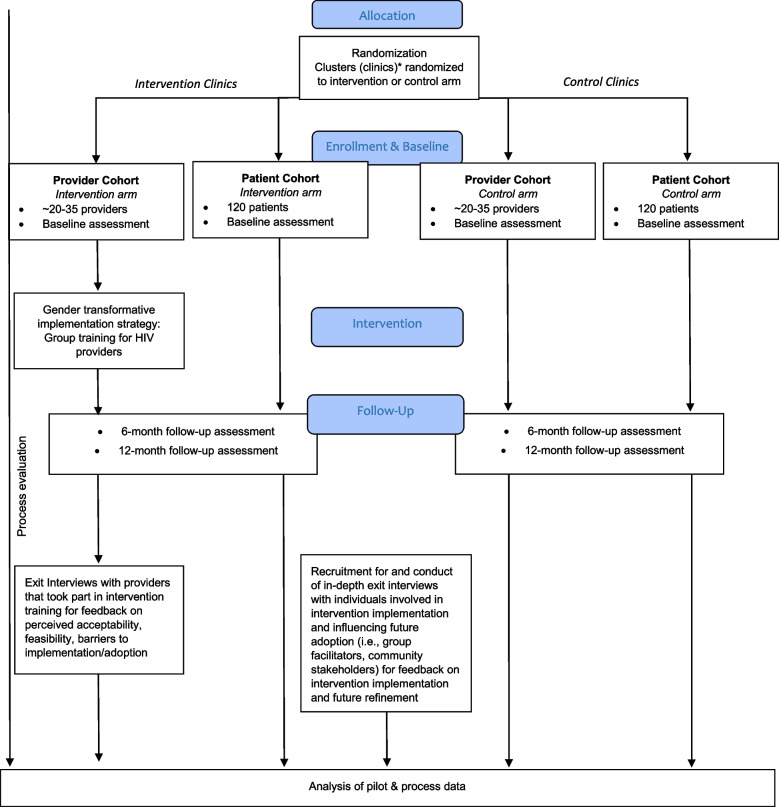
CONSORT diagram. *Note: The two districts (with clinics clustered within them) will be randomized by coin toss

Following Curran et al.’s effectiveness-implementation studies, a hybrid type 3 study is used to test the implementation strategy, gathering information on the intervention and related outcomes [99]. This design is appropriate for clinical interventions such as ART that have strong evidence of effectiveness but are in need of strategies to improve delivery/quality and reduce disparities between groups [27, 99]. The proposed quasi-experimental pre/post intervention design will include at least four sites, determined by recruitment needs. Recruitment will begin in two hospital sites and expand to two additional Health Centre IVs (HCIVs); a staggered approach to recruitment is used so that the study staff and facilitators can focus their efforts on one set of sites before moving to the next sites. The intervention will be implemented at one hospital site, and one HCIV, and the pre/post intervention outcomes will be compared to that of the control hospital and HCIV site (no intervention). Assignment of distrcits will be determined by coin toss. See Fig. 3 below for a visual depiction of the proposed study design.

### Study population

This study will take place in two semi-rural districts in central Uganda. The study population will include two cohorts, including one comprised of HIV care providers and another comprised of HIV patients working/receiving care in the selected facilities. Only providers will receive the implementation strategy; the patient cohort is included to explore whether the intervention strategy’s preliminary effect on provider attitudes/behaviors translates to improvement in patient outcomes.

The provider and patient cohorts will be recruited from the participating clinics. The specific inclusion criteria for each cohort are listed in Table 1. Providers include all healthcare providers or lay workers who provide care/services to HIV patients. Patients include those newly diagnosed and/or initiated on ART and those struggling with treatment adherence, as the implementation strategy will focus on counseling patients in this category, as they are at greatest risk of dropout [100–103].

**Table 1:** Inclusion criteria for the study’s two cohorts: HIV providers and HIV patients

**Provider cohort**	
1. Healthcare providers or lay workers who provide care and/or services to HIV patients at the selected clinics (e.g., medical officers, clinical officers, HIV nurses, midwife, linkage facilitators, counselors)	
2. Fluent in English or Luganda	
3. 18 years of age or older	
**Patient cohort**	
1. HIV infected	
2. Enrolled in HIV care at the clinic of recruitment	
1. Pre-ART (newly diagnosed) or newly initiated on ART (within 1 year) or struggling with treatment adherence, defined in two ways a. Most recent viral load results unsuppressed as assessed through clinic records b. Or self-reported nonadherence	

### Sampling

The goal is to recruit the entire population of providers that work with HIV patients at each of the clinics, which is approximately 20–35 providers per clinic for participation; approximately 35 at each hospital and 25 at each HCIV, totaling to 80–140 providers; the total is an approximation, dependent on the total number of providers per clinic and whether the four-site trial expands to meet patient recruitment targets. With Mildmay Uganda, the research team will generate support for the training first in the broader Health Districts, with medical superintendents and district health officers, through in-person meetings and by garnering their feedback on the intervention through invitations to individual and group meetings with stakeholders to discuss intervention procedures and materials. These stakeholders will be key in the purposive sampling approach used to recruit providers for the study. Although the study aims to recruit all HIV providers at each clinic for participation, any provider can decline to participate. Providers will give written informed consent at the time of enrollment. To mask the randomization of intervention and control, research staff will consent all providers for participation in all aspects of the training intervention, regardless of treatment arm assignment.

Patients will be non-randomly approached when presenting to care by a research assistant based on a review of clinic records. After being referred to the study by a clinician, the research assistant will inform individuals of the study and assess eligibility using an eligibility screening tool. If eligibility is met and the patient would like to participate, the research assistant will obtain written informed consent and collect patients’ contact information (for follow-up reminders) and conduct the baseline questionnaire in the clinic or another agreed upon location.

### Description of the study arms

#### Control

The providers in the district randomized to the control arm will receive no intervention but will receive the standard of care in the clinic.

#### Intervention

The intervention, or implementation strategy, is a multi-session training aimed to improve the quality of care provided to HIV patients. Trained intervention facilitators who are Mildmay expert trainers (health workers experienced in health professional trainings) will lead the training at the intervention clinics. The intervention content delivered is aimed to increase providers’ knowledge, motivation, skills, and empathy to: (1) equitably deliver ART program guidelines (i.e., quality of care) and (2) provide gender-responsive care with a focus on counseling patients newly diagnosed/initiated on treatment.

The intervention integrates and adapts a set of existing strategies intended to inform provider trainings [72], based on a body of research that has shown that increasing provider motivation [73–76], awareness [77–79], skills [80–83], empathy [84–89], and emotional regulation [90–93] can prevent implicit racial and gender biases from affecting clinical judgment and behavior. For the current intervention, these strategies are adapted to increase providers’ knowledge, motivation, skills, and empathy to equitably deliver Ugandan Ministry of Health (MOH) ART program guidelines [104] to men and women patients (e.g., increasing awareness of HIV gender disparities, increasing empathy/skills to counsel men and women’s gendered barriers to care, promoting shared decision-making, promoting patient-centered and gender-sensitive/-specific communication). See Table 2 for an overview of the adapted strategies and Table 3 for an overview of the tentatively planned session content that is built around these strategies (may be subject further adaption before implementation).

**Table 2:** Intervention strategies to reduce gender bias integrated into training content

1	Enhance internal motivation to respond without bias by making providers aware of their own biases and adopt more gender equitable attitudes
2	Increase awareness of societal gender inequity/bias by providing examples of gender bias and evidence of disparities in HIV care
3	Explain bias in the context of societal inequities influencing normal human thought processes
4	Increase perspective taking and empathy related to men and women’s gendered barriers to care via imagery and role-play of patient interactions
5	Encourage partnership building by reframing clinical encounter as consultative decision-making
6	Enhance provider confidence in counseling by increasing awareness of socialized gendered communication and teaching patient-centered communication skills and gender-transformative counseling skills to improve equitable communication with patients
7	Teach emotional self-regulation techniques (e.g., mindfulness) for application in clinical encounters

**Table 3:** Tentative program content

**Session 1: Recognizing and reducing gender disparities in HIV care** *One full-day session*	**What is gender? Understanding gender, gender norms, gender disparities in HIV care** **•** What is gender? Relevant terms & concepts related to gender; the relationship between HIV, gender, and health disparities **Reducing the “HIV threat:” workshop on providing gender-transformative and gender-specific HIV care to improve patient’s HIV care engagement** • Discuss the continuum of gender-responsive health programs • Conduct interactive role-play/case study to identify HIV’s perceived “threat” to men’s and women's roles and respond with gender specific and/or transformative approaches to overcome threats
**Session 2: Understanding how gender dynamics shape clinical interactions** *One full-day session*	**What is bias? Understanding how gender dynamics shape clinical interactions** • What is gender bias? Bias, gender assumptions, stereotypes, and HIV stigma that influence HIV patient care • Workshop in emotional regulation techniques to reduce gender bias in clinical interactions **Communication and power dynamics** *•* Understanding how communication and power dynamics are shaped by gender in the patient-provider relationship *•* Workshop in patient-centered care and communication skills
**Session 3: GBV in HIV care** *~3 h*	Understanding and responding to gender-based violence (GBV) in HIV care* *•* Refresher training to equip health workers with competencies to understand the connection between GBV and HIV and manage GBV survivors/victims in HIV care

Sessions 1 and 2 are delivered in two consecutive days; content is subject to modification before implementation; *content abbreviated and adapted to be HIV focused from the Uganda Ministry of Health's GBV training manual

### Data collection procedures

#### Provider cohort: baseline assessment, 6-month, and 12-month follow-up assessments

Research assistants will conduct baseline, 6-month, and 12-month follow-up assessments with providers. The baseline interviews will be conducted immediately following enrollment and consent or at another agreed upon time and location decided by the participant. Baseline questionnaires will include demographic information and measures to assess provider outcomes, and follow-up interviews will serve as a measure of intervention effects on provider outcomes (see Measures). Each assessment will take approximately 25 min and can take place in person or over the phone through interviewer-administered computer-assisted personal interviewing (CAPI) software. Providers will receive the following compensation for each interview completed: 10,000 shillings (~US $3) (baseline, 6 months) and 18,000 shillings (~US $5) for the final assessment (12 months); amounts were determined appropriate by the local institutional review board (IRB) based on what is standard in the region.

#### Patient cohort: baseline assessment, 6-month, and 12-month follow-up assessments

Research assistants will similarly conduct baseline, 6-month, and 12-month follow-up assessments with patients through interviewer-administered CAPI software in a private space in the clinic or another agreed-upon location upon. Follow-up assessments may be administered via telephone for patient participants not returning to the clinic. Demographic information and measures to assess patient outcomes related to HIV care engagement (e.g., clinic attendance, medication adherence, patient satisfaction) will be collected at baseline immediately following enrollment, with follow-up interviews assessing change in these outcomes. Data will also be extracted at baseline and each follow-up from patient clinic records and directly entered into the computerized survey (i.e., treatment regimen, CD4+ cell count, viral load). Patients will receive the same compensation as the provider cohort: 10,000 shillings (~US $3) (baseline, 6 months) and 18,000 shillings (~US $5) for the final assessment (12 months).

#### Exit focus groups with providers receiving the implementation strategy

HIV providers participating in the training will be invited to participate in exit focus groups after all data collection is complete (6–8 provider per group). The purpose of these focus groups is to assess the perceived acceptability, feasibility, barriers to implementation/adoption, and other measures of implementation among providers who participated in the training. Focus groups will be facilitated by a trained, experienced focus-group facilitator/qualitative interviewer and will last approximately 90 min. Focus-group facilitators will follow a focus-group question guide. They will be audio recorded with participants’ permission and transcribed after completion.

#### Exit interviews with intervention facilitators and key stakeholders

Semi-structured qualitative interviews will be held with the trainers (intervention facilitators) who implement the training intervention, any provider not able to attend focus groups, and with approximately four Mildmay leaders in charge of the integration of courses into Mildmay’s training programs and other district health leadership. Exit interview guides for intervention facilitators will elicit challenges and successes in implementing the intervention. Participants will be recruited directly by the project coordinator for participation. Mildmay leadership and other district stakeholder interviews will assess barriers and facilitators to the implementation and future adoption of the training. They will be referred to the study by the study’s researcher partners at Mildmay Uganda. The audio-recordings will be transcribed after completion, and interviews conducted in Luganda will be translated to English.

#### Data collection to assess intervention fidelity

To assess fidelity, the first group training sessions will be observed by trained research staff with a checklist created to assess the delivery of content. These observed sessions will allow the investigators to give immediate feedback for improvement during implementation. In addition, all sessions will be audio-recorded, 20% of which will be randomly selected at the end of the study to be qualitatively analyzed to assess Mihalic’s [105] fidelity dimensions.

##### **Measures (aim 1): explore CFIR barriers and facilitators to implementation and adoption**

The primary aim of this pilot trial is to assess the implementation strategy’s acceptability and feasibility (primary outcomes), and barriers and facilitators to the implementation and future adoption of the training (secondary outcomes) with the goal of improving the sessions before larger scale-up and evaluation. The data that will be collected for this aim includes the qualitative methods described under “data collection procedures,” including exit interviews, exit focus groups, and procedures to assess intervention fidelity. The primary outcomes explored through this qualitative data are guided by CFIR, as outlined in Table 4, which is organized by CFIR domain.

**Table 4:** CFIR [28] domains and constructs [106, 107] explored as barriers and facilitators to the implementation and adoption of the intervention (aim 1)

CFIR domain and constructs	Sample^a^
CFIR domain: Intervention characteristics	
**Intervention source**: Perception of key stakeholders about whether the intervention was externally or internally developed	Trainers, stakeholders
**Complexity/barriers**: Perceived difficulty of/barriers to implementation/application of content into clinical care	Trainers, providers, stakeholders
**Appropriateness**: The perceived fit of the intervention for the setting and to improve care quality and patient outcomes	Trainers, providers, stakeholders
**Intervention fidelity**: Was the intervention implemented as intended?	Assessment of recorded sessions
CFIR domain: Outer setting	
**Patient needs**: The extent to which patients’ gendered barriers and facilitators to care are accurately known and prioritized	Trainers, providers, stakeholders
CFIR domain: Inner setting	
**Relative priority**: Perceived importance of implementation within the clinic and broader health system	Trainers, providers, stakeholders
**Culture**: Organizational norms, values, and basic assumptions specific to the following: (a) innovations in care and (b) gender equity	Trainers, providers, stakeholders
CFIR domain: Individual characteristics	
**Knowledge and beliefs about the intervention**: Attitudes towards and value placed on the intervention, including perceptions on *acceptability* (satisfaction with intervention) and *feasibility* (can intervention be carried out within clinic/broadly)	Trainers, providers, stakeholders
**Self-efficacy**: Beliefs in own ability to execute implementation goals (trainers) and training content (providers)	Trainers, providers
**Other personal attributes**: Gender, age, and other traits	Trainers, providers

^a^Data collection methods: *providers*, focus-group discussions; *trainers*, in-depth interviews; *stakeholders*, key informant interviews

##### **Measures (aim 2): assess the feasibility of trial procedures**

The second aim of this pilot trial is to gather information on the feasibility of trial procedures. Process data will be collected throughout the trial to assess the outcomes within this aim, which are listed in Table 5.

**Table 5:** Process evaluation measures for feasibility of trial procedures (aim 2)

Outcome	Measures
*Clinic mobilization*	Percent of clinics assessed for participation that are found eligible and reasons for ineligibility, collected through process data/study records; Percent of eligible clinics invited for participation that accept and reasons for decline, collected through process data/study records
*Recruitment*	Percent of individuals enrolled per month in each cohort and reasons for decline, collected through process data/study records
*Retention*	Treatment-specific retention rates for each cohort through 12-month follow-up and reason for dropout, collected through process data/study records observation and exit interviews
*Assessment process*	Percent of planned assessments completed and duration of assessments collected through process data collection
*Fidelity*	Degree to which intervention was implemented as prescribed in the protocol, measured through the audio-recording and analysis of randomly selected sessions (as described in text)

##### **Measures (exploratory aim 3): explore the potential effectiveness of the implementation strategy on patient and provider outcomes**

The quasi-experimental controlled trial will be used to gather preliminary evidence on the implementation strategy’s potential effectiveness through 12-month follow-up on patient outcomes related to HIV care engagement and quality of care, as well as provider outcomes related to change in knowledge, attitudes, and motivation to provide quality, gender-responsive care. While this pilot study is not powered to detect statistically significant changes in all outcomes, the collection of all planned outcome measures can determine the preliminary effects of the intervention while helping to determine the feasibility and acceptability of study measures and data collection procedures (as specified in aim 2). See Table 6 for an overview of the exploratory outcome measures.

**Table 6:** Exploratory outcome measures used to pilot the acceptability and feasibility of measurement tools and procedures and the intervention’s preliminary effect on provider and patient outcomes (exploratory aim 3)

**Provider cohort**		
**Exploratory primary outcome measures**	**Data collection procedures/measures**	**Time frame**
*Gender awareness*	Gender awareness is measured with the Adapted Nijmegen Gender Awareness in Medicine Scale (N-GAMS) [108], developed for medical personnel. The sub-scales have good content validity and reliability (Cronbach’s *α* = 0.73–0.86) in developed settings [108]. Two subscales will be adapted for the present study to measure: *• Attitudes towards gender sensitivity*: This scale measures attitudes towards gender sensitivity in healthcare, with items that measure agreement on the perceived importance and perceived outcomes of gender-sensitive care, adapted by the study team to be HIV specific *• Gender stereotypes towards patients*: Items originally developed to measure gender stereotypes about patients in healthcare settings will be adapted by the study team to be specific to the cultural context of Uganda, including common stereotypes and bias specific to gender and HIV	Baseline, 6 and 12 months
*Competence for gender-responsive care*	Competence for gender-sensitive care will be measured through an adaption of Saha et al.’s Self-Rated Cultural Competence Instrument for Primary Care Providers that assesses awareness, perceived importance, motivation, and skills to provide culturally competent care [109]. For the current study, the scale is adapted to be specific to competence for gender-responsive HIV care. The original scale has items mapping onto specific domains, adapted for our study, including the following: *• Awareness of societal gender inequities*: The original scale included items to assess the provider attitudes on disparities in health and healthcare. For the present study, items will be adapted to assess providers’ agreement with statements on societal-level gender inequities that favor men and disadvantage women *• Awareness of gender disparities in HIV care*: Within the original scale’s domain of disparities in health and healthcare, items will be adapted to measure providers’ knowledge of HIV gender disparities *• Gender-sensitive care/counseling skills and behavior*: The original items developed to assess the level in which providers engage in gender-responsive care behavior and their perceived self-efficacy or skill for delivering gender-responsive care will be adapted to be HIV specific	Baseline, 6 and 12 months
***Exploratory secondary outcome measures***	**Data collection procedures/measures**	**Time frame**
*Communication self-efficacy*	An adapted version of the self-efficacy questionnaire (SE-12) for provider communication will be used to assess communication self-efficacy, adapted to be gender specific	Baseline, 6 and 12 months
*Gender equitable attitudes*	Gender equitable attitudes will be measured with the Gender Equitable Men scale [110] validated in Tanzania and Ghana [111], with a Cronbach’s *α* = 0.79–0.88 in African settings [111–114]	Baseline, 6 and 12 months
*Empathy*	Provider empathy for patient experiences will be measured from an adapted version of the Jefferson scale of physician empathy, which has been adapted for HIV care previously [115, 116]	Baseline, 6 and 12 months
*Emotional regulation and stress reduction techniques*	Providers’ use of emotional regulation and stress reduction techniques, such as breathing exercises, sense soothing, tension release, attention shifting, and positive reframing, will be measured through items adapted from the Mindful Self-Care Scale (MSCS) and the Brief COPE [117, 118]	Baseline, 6 and 12 months
**Exploratory primary outcome measures**	**Data collection procedures/measures**	**Time frame**
*ART adherence*	Measured by self-report, through the Adult AIDS Clinical Trials Group (AACTG) scale’s [119] 4-day adherence recall questions; demonstrated good construct validity in Uganda [120], strong correlations with viral load [121], and moderate correlations with electronic adherence monitoring [122]	Baseline, 6 and 12 months
**Patient Cohort**		
***Exploratory secondary outcome measures***	**Data collection procedures/measures**	**Time frame**
*Short-term retention in care*	Operationalized in two ways, collected through patient clinic records, and triangulated with self-report *• Missed visit count*: Number of missed visits accrued (count measure) based on scheduled visits determined by MOH clinical guidelines *• Visit adherence*: Proportion of kept visits/scheduled visits (kept + missed visits) (continuous measure, range = 0.0–1.0)	Baseline, 6 and 12 months
*Viral load*	Collected from patient clinic records* and confirmed with self-report, viral load will be operationalized in two ways *• Change in viral load*: Change in viral load will be measured with statistically significant reductions defined as a threefold, or a 0.5 log_10_ copies/mL, change [123] *• Viral load suppression*: Viral load suppression will be defined as HIV RNA < 200 copies/mL	Baseline, 12 months
*Quality of communication*	Patient’s perceptions of the quality of communication with their HIV providers will be measured through two scales *• Quality of communication*: Developed by Wilson et al. [124] for HIV populations, items measure the perceived quality of general health communication from HIV providers, asking patients to rate the quality of their HIV providers in communicating general health information and in providing HIV-specific information *• Quality of adherence dialogue*: Patients’ perceived quality of provider communication specific to ART adherence will be measured from items adapted from Schneider and colleagues [125]	Baseline, 6 and 12 months
*Participatory decision-making*	Participatory decision-making style of HIV providers, or how active of a role patients perceive they have in their healthcare decisions, will be measured with Kaplan’s 7-item scale [126]	Baseline, 6 and 12 months
***Exploratory secondary outcome measures***	**Data collection procedures/measures**	**Time frame**
*Overall satisfaction with care*	The GHAA Consumer Satisfaction Survey to measure overall satisfaction with care adapted to focus specifically on HIV care will measure patient satisfaction with HIV care [127]	Baseline, 6 and 12 months
*Provider trust*	Provider trust will be measured with items from the Primary Care Assessment Survey by Safran and colleagues [128]	Baseline, 6 and 12 months
*HIV stigma*	General HIV stigma and HIV stigma from healthcare providers will be measured using Earnshaw’s HIV stigma framework scale [129], which measures anticipated, enacted, and internalized HIV stigma	Baseline, 6 and 12 months

Notes: *Clinics follow MOH guidelines for routine viral load testing; viral loads are taken as part of routine care after 6 months of ART and every 12 months thereafter (or 6 months if detectable). Since patients will be newly initiated on treatment or unsuppressed, they should have had a viral load recently taken or will be eligible for viral load at baseline or 6 months and 12 months later

### Data analysis approach

#### Process evaluation using qualitative and process data (aims 1 and 2)

A mixed-methods approach will be used to assess the acceptability and feasibility of intervention content and trial procedures, using both qualitative and quantitative data. For aim 1, feasibility, acceptability, and barriers and facilitators to implementation/future adoption, exit focus groups and interviews will be audio-recorded, translated, transcribed, and analyzed using a thematic analysis approach. The investigative team will devise a coding scheme a priori based on CFIR. Trained research assistants will independently code the data under the supervision of the primary investigator. The team will review codes for consistency, refine the coding scheme as needed, and identify major themes, repeating this process until consensus is reached. For aim 2, process data to assess the feasibility of trial procedures will be analyzed using descriptive analysis methods and mixed with qualitative data when possible (e.g., quantitative data on retention can be paired with qualitative data on potential reasons for participant drop out). Qualitative findings will be mixed with quantitative process data during the presentation and interpretation of results [98].

#### Quantitative data and pilot intervention efficacy (exploratory aim 3)

As a pilot intervention trial, the study’s exploratory aim to gather preliminary effects of the intervention on patient and provider outcomes will be achieved through mainly descriptive analyses. Using SPSS v.27, baseline characteristics of providers and patients will be summarized by the different clinics to assess comparability of the study groups. We will use frequencies and descriptive statistics to describe the study’s exploratory primary and secondary outcomes across the two study arms across the two cohorts: *patient outcomes*: ART adherence and secondary outcomes and *provider outcomes*: gender awareness, competence for gender-responsive care, and secondary outcomes. We will look at the mean difference between the two arms in these outcomes and use the magnitude of the difference (if present) to inform the design of a future, fully powered trial cluster randomized controlled trial.

#### Sample size justification

The sample size is based on guidelines for stage 1b studies (feasibility and pilot testing of new behavioral interventions), which suggest 15–30 participants per cell [130]. Thus, 120 patients and ~20–35 providers per clinic (accounting for variance and transience in staff) fit within these guidelines and allows for the feasible pilot implementation of the training at two clinics. G*Power (assuming power = 0.80, *α* = .05) was used to confirm a sample of 100 patients per condition (estimating 17% attrition from *n* = 120) would detect a moderate effect size (*d* = 0.4) in ART adherence (based on systematic reviews of ART adherence interventions inclusive of sub-Saharan African studies) [131–133].

### Ethics

All protocols have been approved by the IRB at Makerere University and the University of Texas at San Antonio. In addition, the research team will continue to consult with Mildmay Uganda and participant Districts’ leadership to ensure that the measures, consent procedures, and proposed intervention are acceptable and appropriate to the community. Finally, the primary investigators and study staff will review the progress of the research and available data to identify the occurrence of adverse events and appropriately respond to any such events should they occur. IRB-approved informed consent procedures and forms will be obtained for participation in the providers’ intervention and assessments, patient assessments, and exit interviews with intervention facilitators and Mildmay stakeholders to ensure that participants are fully aware of any possible risks and benefits. Participation in the research is voluntary, as is answering each question on the various questionnaires and participating in group discussions and training activities during the training sessions with group facilitators. Employment, treatment, and care received at the participating clinics will not be affected by a lack of participation in the study, and participants will be reminded that their participation in this research can be terminated at any time. Research staff will attend training sessions and receive ongoing supervision in areas related to ethical conduct, confidentiality protection, and other topics related to human participant protection. We expect the benefits of participation in this study to exceed the risks. The main potential risks of the study are summarized in Table 7 with details on strategies that will be employed to minimize each risk.

**Table 7:** Overview of ethical considerations: potential risks of participation and planned strategies for risk mitigation

Risks	Safeguards for risk mitigation
**Potential for breaches in confidentiality related to collected data**	(1) The use of unique identifiers instead of medical identification/record numbers or participant names The storing of the lists that link the participants to their unique identifiers in locked, secure locations (2) The use of password protection for all data collected and/or stored electronically (3) Training all study staff in the importance of and procedures for protecting participants’ confidentiality, including the use of a signed confidentiality agreement
**Potential to experience discomfort while discussing sensitive information during interviewer-administered computerized questionnaires and group intervention sessions**	(1) IRB-approved consent forms will convey that the survey portion of the research project and group sessions involves sensitive topics (2) The ability to skip any questions that make one uncomfortable and to withdraw from the study any time (3) Training of study staff to approach sensitive topics in a culturally appropriate and nonjudgmental way
**Potential unintended negative consequences on participants in the intervention (e.g., conflict between colleagues)**	(1) Inform participants of potential risks in the informed consent process (2) Training of group facilitators to create a safe space for open discussion at the start of each session (3) Training of group facilitators in strategies to approach discussions that challenge gender norms carefully

## Discussion

This protocol describes the pilot and evaluation of an implementation strategy to increase providers’ capacity to provide equitable and gender-responsive treatment and counseling to HIV-infected men and women. Delivered to HIV providers, this group training integrates a gender-responsive approach with adapted evidence-based strategies to reduce biases and increase gender equitable attitudes. The implementation strategy aims to improve ART adherence/retention interventions by improving different elements of the patient-provider relationship.

This study will provide training to health workers on the equitable delivery of ART counseling and innovative gender-responsive ways to increase men and women’s engagement in HIV services. We expect that the overall risk to benefit ratio will be favorable to individuals participating in the study. The risks associated with completing the measures and participating in the implementation strategy have been minimized via informed consent procedures, procedures for maintaining confidentiality, and extensive training of group facilitators and research staff. Given the potential benefits to participants, and the considerable individual and public health benefit potential of the knowledge to be gained in this research, the minimal risks involved are judged to be reasonable.

The specific benefits that may be gained among providers through the development and implementation of this intervention include increased knowledge of and motivation to reduce gender bias and disparities in care and address patients’ gendered barriers to care; improved communication skills, joint decision-making, and equity with patients; skills in gender-transformative counseling among providers; and increased gender equitable attitudes among providers. By increasing gender equitable attitudes among providers and reducing gender bias in the provision of care, it is anticipated that this study will improve providers’ interactions with patients, which will benefit patient health outcomes. This includes patient viral load suppression, improved through patients’ increased satisfaction with services, clinic attendance, and ART adherence. Given that the training focuses on issues for patients newly diagnosed and initiated on treatment, the provider training has the potential to improve care for patients at greatest risk of dropout and thus can engage patients in care at a critical point in the treatment cascade. Importantly, viral load suppression and engagement in HIV care have significant health benefits for people living with HIV and on a population level may reduce HIV incidence by reducing the viral load of those living with HIV to noninfectious levels. By focusing on issues related to gender, this implementation strategy could also reduce gender disparities in HIV outcomes between men and women. While we aim to improve both men and women’s engagement in care through this implementation strategy, engaging men in HIV care is especially needed in Uganda and similar settings. This implementation strategy could help to address men’s specific barriers and bring their levels of engagement closer to those achieved for women.

This implementation strategy represents an important attempt to develop strategies that are effective in improving the quality and effectiveness of ART counseling in the context of Uganda, to engage providers in equitable decision-making and communication with patients, to increase capacity for gender-sensitive counseling, and to generate facility support for gender equity in HIV care. This study could also provide data on the potential use of the gender-transformative training sessions with providers as a scalable approach to reduce gender disparities in HIV care, which would have important implications for Uganda’s national scale-up of efforts to improve patient viral load suppression and engagement in HIV care and reduce gender gaps in HIV outcomes between men and women.

### Limitations

We chose to randomize by clinic, and not by individual, because intervention components are delivered at the clinic level. As a pilot trial, we can only randomize a small number of clinics, which could introduce confounding variables given too few clusters. However, this pilot can inform the procedures for a larger cluster randomized controlled trial after acceptability and feasibility are established. Another potential limitation includes the potential for contamination between treatment conditions. This is mitigated by choosing clinics that are greater than 1 h in travel time apart making it unlikely that patients would attend both clinics for care. Participants and interviewers will be told that participants are randomized to receive some, all, or none of this content in order to attempt to mask the true goal of the study. Finally, we include viral load as an exploratory outcome, relying on clinic record data for its measurement. Although the clinics conduct routine viral load collection following Uganda MOH guidelines, this outcome may be subject to missing data. In addition, as a pilot study, we have limited power to detect differences in viral load; however, trends towards change in viral load and related determinants will provide evidence to support a future, fully powered trial. Progression criteria to help determine the next step beyond this pilot trial are included as supplementary material. Following Thabane et al.’s recommendations [134], the progression criteria will be used to determine whether the next step is progression to a larger, fully powered controlled trial without modification to the protocol, progression without modifications but with close monitoring, progression with modifications, or no progression if deemed not feasible/acceptable even after modifications. The progression criteria focus on the key outcomes related to the acceptability of the intervention, feasibility of recruitment, fidelity of intervention implementation, and feasibility of outcome measurement.

## Conclusion

If the pilot implementation strategy is found acceptable and feasible and shows preliminary effects on ART adherence and other favorable patient and provider outcomes, the findings may be used for implementation of an innovative gender-transformative training which could be institutionalized and widely disseminated in Mildmay Uganda’s existing medical training programs and at the national level. This study could also provide data on the potential use of the gender-transformative training sessions with providers as a scalable approach to reduce gender disparities in HIV care, which would have important implications for Uganda’s national scale-up of efforts to improve patient viral load suppression and engagement in HIV care and reduce gender gaps in HIV outcomes between men and women. This implementation strategy also has the potential to be generalizable to settings where gender norms amplify the HIV epidemic and could be adapted to improve implementation of other aspects of the HIV prevention-care continuum (e.g., pre-exposure prophylaxis).

## Supplementary Information

**Additional file 1.** Progression Criteria.

## Data Availability

Data sharing is not applicable to this protocol article as no datasets were generated or analyzed.
