# Supplementary material for: Protocol for the pilot quasi-experimental controlled trial of a gender-responsive implementation strategy with providers to improve HIV outcomes in Uganda
Source: Pilot Feasibility Stud. 2022 Dec 23;8:264. doi: 10.1186/s40814-022-01202-0 (PMC9783690; doi:10.1186/s40814-022-01202-0)
Supplement: Supplementary file 1 — Additional file 1. Progression Criteria. [file 40814_2022_1202_MOESM1_ESM.docx]

**Progression Criteria**

| **Outcomes** | **Criteria** | **No modifications** | **Consider modification and closely monitor** | **Modifications needed to progress** | **Stop (not feasible/acceptable)** |
| --- | --- | --- | --- | --- | --- |
| **Acceptability of intervention** | % of participants reporting being satisfied or very satisfied with the intervention sessions | 90-100% | 80-89% | 50-89% | <50% |
| **Feasibility of recruitment** | % of eligible participants that can be recruited | 75-100% | 70-74% | 50-69% | <50% |
| **Fidelity of intervention** | % of all intervention sessions delivered to participants | 89-100% | 72-88% | 50-71% | <50% |
| **Feasibility of outcome measurement** | % complete follow-up in enrolled participants | 91%-100% | 79-90% | 60-78% | <60% |

Following Thabane et al.,^1^ the information above will be used to determine one of the following outcomes for the pilot study:

1. Stop - main study not feasible/acceptable;
2. Continue, but modify protocol - feasible/acceptable with modifications
3. Continue without modifications, but monitor closely and consider modifications to improve protocol – feasible/acceptable with close monitoring; modifications may improve feasibility/acceptability
4. Continue without modifications - feasible/acceptable as is

^1^ Thabane L, Ma J, Chu R, et al. A tutorial on pilot studies: the what, why and how. BMC Med Res Methodol. 2010;10:1. Published 2010 Jan 6. doi:10.1186/1471-2288-10-1
